# A Relational Identity-Based Solution to Group Polarization: Can Priming Parental Identity Reduce the Partisan Gap in Attitudes Toward the COVID-19 Pandemic

**DOI:** 10.1177/10755470211036676

**Published:** 2021-12

**Authors:** Chen Zeng

**Affiliations:** 1 Temple University, Philadelphia, PA, USA

**Keywords:** COVID-19, parental identity, partisan identity, polarization

## Abstract

This study explores the influence of both group identity (e.g., partisan identity) and relational identity (e.g., parental identity) on beliefs and attitudes toward the coronavirus disease 2019 (COVID-19) pandemic. Results from a between-subject randomized survey experiment suggest that partisans are motivated to process factual information about COVID-19 through a partisan lens. However, priming parental identity can reduce partisan polarization over risk perceptions, policy support, and precautious behaviors. These findings demonstrate the need to incorporate relational identity into identity-based science communication research and offer a relational identity-based strategic communication solution to partisan gaps in responses to COVID-19.

People hold polarized attitudes toward a range of scientific issues (e.g., Hart & Nisbet, 2012; Scheufele et al., 2009). Scholars often examine attitude polarization through a lens of group dynamics and have long acknowledged the role of group-based identity, particularly partisan identity, in shaping public opinion on scientific issues and policy preferences (e.g., Dunlap et al., 2016; Hart et al., 2015). Social identity theory (Tajfel, 1981) and self-categorization theory (Turner, 1985) are used as the major theoretical frameworks to understand the influence of partisan identity on message processing and individuals’ beliefs and attitudes toward scientific issues. In addition, group identity can generate motivated reasoning (Kunda, 1990) and identity-protective forms of cognition (Kahan et al., 2007) in information processing.

With very few exceptions, researchers studying the relationship between social identity and attitudes exclusively focused on group-based identities. There is no doubt that group-based social identity can have powerful impacts on people’s beliefs and attitudes within the sociopolitical context. However, social identity is multifaceted (Deaux, 2001). One’s identity is not solely determined by group membership but also influenced by interpersonal relationships (Brewer & Gardner, 1996). Brewer and Gardner (1996) used the term *relational identity* to describe the self-concepts people derive from interpersonal relationships. Relational identities, such as family roles and friendship, are often emphasized when individuals are asked to list their most important identities (Reid & Deaux, 1996). Despite the importance of relational identity in people’s lives, we know surprisingly little about the role of relational identity in information processing and attitude formation toward scientific issues.

Similar to group identity, relational identity provides a lens through which the world is perceived and attitudes are formed (Reid & Deaux, 1996). Based on interpersonal communication literature, relational identity should provide a meaningful basis on which to process scientific information because it can trigger altruistic motivation (Baumeister & Leary, 1995). It is, therefore, helpful to consider how relational identity can help counter polarization resulting from group memberships because science communication scholars and practitioners often seek to identify message strategies with which to effectively communicate scientific facts and decrease attitude polarization.

This study incorporates relational identity into identity-based attitude polarization studies in science communication and explores the influence of both group identity (e.g., partisan identity) and relational identity (e.g., parental identity) on beliefs and attitudes toward the COVID-19 pandemic, which is an ongoing issue that presents humanity with extraordinary challenges. In addition, this study offers a relational identity-based strategic communication solution that could potentially decrease the influence of partisan identity on beliefs and attitudes toward politicized issues by investigating the moderating role of the parental identity prime in the relationship between partisan identity and COVID-19 attitudes.

## Distinguishing Between Group Identity and Relational Identity

Identity refers to an individual’s sense of self (Brewer & Gardner, 1996). People hold multiple identities (e.g., a daughter, mother, African American, and Democrat), and these identities are not fixed but rather dynamic and flexible (Jenkins, 2014; Oakes et al., 1991). The salience of different identities might, in turn, prompt different emotional, cognitive, and behavioral responses (Oakes et al., 1991). Brewer and Gardner (1996) identified three levels of identity: individual (or personal), relational (or interpersonal, role), and group (or collective, social). Individual identities are social identities that people use to define themselves as certain types of people. Relational identities are identities derived from interpersonal relationships (Brewer & Gardner, 1996). Group identities speak to the perception of self that is derived from group memberships (Brewer, 2001).

Although both relational identity and group identity are social extensions of the self, they are theoretically different constructs (Brewer & Gardner, 1996). Distinguishing between the two concepts is necessary to examine their impacts, respectively, and how they might interact in information processing and attitude change. First, the bases of relational identity and group identity are different. Relational identity is based on the premise that individuals are interdependent. It explicitly involves a connection with a relational other and captures one’s identity when relating to others (Andersen & Chen, 2002; Brewer, 2001). Group identities, however, do not require interpersonal relationships among group members. As Brewer (2001) put it, group identities are “not forged from interpersonal relationships between and among individual group members, but rather from common ties to a shared category membership” (p. 119). In short, relational identity is relatively unique to an individual because it is derived from the interaction between the individual and the relational other while group identities are shared among group members (Andersen & Chen, 2002).

Second, different levels of identity are associated with different basic motivations. Group identity is often associated with the motives of maintaining group status and following group norms. As Sluss and Ashforth (2007) explained, on the group level, “the basic motivation is the welfare of the collective, placing a premium on common fate, cohesion, and group norms” (p. 10). On the relational level, the basic human motivation is the partner’s well-being, and “self-esteem derives from fulfilling one’s role-relationship obligations” (Sluss & Ashforth, 2007, p. 9). In other words, relational identity is often associated with the motive of protecting the other party in a relationship and maintaining the relationship (Brewer & Gardner, 1996). Research has shown that when interpersonal relationships drive identity, there is a shift from the motivation for the group’s welfare to the motivation for the benefit of the other (Baumeister & Leary, 1995). In sum, relational identity and group identity involve different foci and motivational bases.

## Partisan Identity and Attitudes Toward the COVID-19 Pandemic

The group identity that has received the most scholarly attention is partisan identity. Past research contains extensive evidence showing that partisan identities motivate individuals to process information in a biased manner and have great impacts on individuals’ beliefs, attitudes, and behaviors (Kahan et al., 2007). In the field of science communication, scholars have recognized the powerful role that partisan identity plays in shaping attitudes toward a range of scientific issues, such as climate change (Pearson & Schuldt, 2015), biomedical research (Nisbet & Markowitz, 2014), fracking (Veenstra et al., 2016), and nanotechnology (Kahan et al., 2009), among others.

Motivated reasoning and identity-protective cognition are two key interrelated theories that can be applied to understanding the role that partisan identity plays in the processing of messages and formation of attitudes. Motivated reasoning refers to the tendency to assess or evaluate information in a biased manner when individuals are motivated to achieve certain goals (Kunda, 1990). According to motivated reasoning, people often do not judge information or evidence on the basis of accuracy. Rather, they are likely to be motivated by directional goals and tend to interpret information to be directionally consistent with their prior beliefs and attitudes (Kunda, 1990). Protecting one’s identity is a directional goal. As a result, identity cues trigger directional motivated reasoning by encouraging people to process factual information in the direction that is in line with their prior attitudes, as dictated by their group memberships (Kahan et al., 2007). Kahan et al. (2007) developed the identity-protective cognition thesis to describe this process. The basic assumption in identity-protective cognition is that people are motivated to maintain and protect their identities (Kahan et al., 2007). Accordingly, people tend to access information or construct beliefs in ways that reinforce their senses of who they are or their group memberships (Kahan et al., 2007).

In the context of COVID-19, this study argues that partisan identity is a driver of risk perception, policy support, and self-reported precautious behaviors associated with the COVID-19 pandemic. COVID-19 was first detected in Wuhan province, China, in early November 2019 (Centers for Disease Control and Prevention [CDC], 2020b). The World Health Organization (WHO, 2020) declared the outbreak as a pandemic on March 11. By December 10, 2020, over 15.2 million cases of COVID-19 had been reported in the United States, and over 286,000 Americans have died as a result of COVID-19 (CDC, 2020a). Slowing the spread of the virus during the pandemic required collective efforts, such as social distancing and mask-wearing in public.

Although the COVID-19 pandemic is a global health crisis, it has become a partisan issue in the United States (Hart et al., 2020). Research examining partisan gaps in response to COVID-19 showed that Americans were divided along party lines concerning their responses to the pandemic, especially in the early stages (Hart et al., 2020). For example, Democrats were found to have higher levels of risk perception about the pandemic than Republicans (Painter & Qiu, 2020). They were also found to be more supportive of government restrictions and more adaptive to behavioral changes in light of the pandemic than others (Evans & Hargittai, 2020). Using large-scale geotracking data, Gollwitzer et al. (2020) found partisan gaps in physical distancing behaviors, which led to relatively higher COVID-19 infection and fatality growth rates in Republican-leaning counties.

The political divergence in COVID-19 attitudes and behavioral intentions may arise from the different sets of values embraced by Democrats and Republicans (Chan, 2021). One framework based on which to understand how partisanship has affected attitudes toward COVID-19 is moral foundations theory (MFT; Graham et al., 2009; Haidt, 2012). According to MFT, individuals rely on moral foundations, such as caring, authority, and liberty, to make social and political decisions. Past research has shown that MFT is closely tied to partisan ideological reasoning (Graham et al., 2009; Haidt, 2012). For example, political conservatives are focused on the moral foundations of individual liberty, loyalty, and authority, while liberals prioritize caring and fairness (Day et al., 2014; Graham et al., 2009). In the context of COVID-19, it is conceivable that Republicans, who value individual liberty more than Democrats, are less likely to support and comply with restrictive policies (Bazzi et al., 2021; Wnuk et al., 2020). The partisan divide in the public’s early reaction to the crisis can also be attributed to the divergent cues sent by Democrat and Republican leaders who are trusted ingroup elites, as well as those sent in politicized COVID-19 news coverage (Green et al., 2020).

This study hypothesizes that partisan identity is a driver of risk perception, policy support, and self-reported precautious behaviors associated with the COVID-19 pandemic. It is also expected that partisan differences would emerge, even in the absence of partisan cues because of the politicization and polarization in the initial COVID-19 news coverage (Hart et al., 2020). Hypothesis 1 is stated formally as follows:

*Hypothesis 1 (H1):* Partisan identity will influence risk perceptions about COVID-19 (H1a), support for restrictive policies to control the outbreak (H1b), social distancing (H1c), and mask-wearing (H1d), such that relative to Democrats, Republicans will have lower levels of risk perception, policy support, social distancing, and a lower likelihood of wearing face masks.

## Parental Identity and Attitudes Toward the COVID-19 Pandemic

Although numerous studies have examined the powerful role of group identity salience in influencing individuals’ policy preferences, the impact of relational identities is understudied. Interpersonal relationship literature provides insights into the potential role of relational identity salience in message processing and attitude formation. The concept of relationships is at the core of interpersonal communication studies. Interpersonal relationships essentially involve mutual concern for the interests and outcomes of both parties (Baumeister & Leary, 1995). The mutual concern for the other person in a relationship can elicit altruistic motivation, which is the motivation to benefit the other (Batson, 1994). Accordingly, relational identity cues in messages should provide incentives for individuals to benefit those they care about. The relational identity examined in this study is parental identity. Parenthood plays a fundamental and pervasive role in people’s lives. The parental role is commonly listed as one of the most important identities and perceived to be relatively more important than other life roles, such as those of partner or friend (Cowan et al., 1991; Reid & Deaux, 1996). As such, the role parental identity plays in shaping attitudes is worthy of study.

Political socialization is the predominant framework upon which to understand the political dynamics of parenthood, especially the impact of having children on sociopolitical attitudes. According to socialization theory, parenthood is an important agent of political socialization among adults because it can bring about changes in adults’ political outlooks and priorities (Jennings & Niemi, 1974). As Elder and Greene (2008) explain, “having a child brings about a salient new social role as a mother or father, which brings with it considerable responsibilities, worries, and psychological demands” (p. 121). Several studies within socialization literature have examined the role of parenting experience in shaping how individuals think about and act in the political world. For example, research showed that motherhood experiences increased mothers’ engagement and participation in family-centered issues such as social welfare programs (Sapiro, 1983) and education policies (Jennings & Niemi, 1974). In another study, Klar (2013) showed that parental identity provided powerful cues that affect message processing and policy support about social services spending.

In the context of the COVID-19 pandemic, it is reasonable to expect that parents will have distinctive attitudes on issues that affect their children’s lives. Care and concern for one’s children lie at the center of parental identity. Although children may experience less severe illness from COVID-19 than adults, the virus still poses threats to children (CDC, 2020c). The numbers of children who tested positive and were hospitalized for COVID-19 have increased since the beginning of August 2020 (Maxouris, 2020). Doctors also linked the severe “multisystem inflammatory syndrome” cases that appeared and increased in young children to coronavirus (Willis et al., 2020). As such, when an individual’s parental identity is made salient, parental concerns about the health and well-being of children also become salient, which can further affect attitudes toward the COVID-19 pandemic. Therefore, the following hypothesis is proposed:

*Hypothesis 2 (H2):* Participants in the parenthood prime condition will show higher levels of risk perceptions (H2a), greater support for restrictive policies to control the pandemic (H2b), greater intentions to practice social distancing (H2c), and greater intentions to wear masks in public places (H2d) than those in the control condition.

Differences in the effects of parenthood on sociopolitical attitudes are frequently found along gender lines (Elder & Greene, 2008). Examining men and women separately helps us understand how parenthood may affect men and women differently. Traditionally, women are considered to be primary nurturers and caregivers. Studies have found that women are more committed to the roles of parenthood and are much more likely to identify motherhood as a primary identity (see Arendell, 2000). In addition, mothers spend significantly more time with their children than fathers do (Elder & Greene, 2008). These findings imply that the parenthood prime might affect men and women differently. Therefore, the following hypothesis is proposed:

*Hypothesis 3 (H3):* In the context of the COVID-19 pandemic, gender is a contributory moderator of the relationship between the parental identity prime and outcome variables including risk perceptions (H3a), policy support (H3b), intentions to practice social distancing (H3c), and intentions to wear face masks (H3d), such that the effects of the parental identity prime are stronger for women than men.

## The Role of Parental Identity in Reducing Partisan Polarization

Previous sections have discussed the impacts of group identity and relational identity cues on information processing and attitude changes. The possible interplay between them is still unclear. One goal of this study was to examine whether the parenthood prime would attenuate the powerful effects of partisan identity on parents’ attitudes toward the COVID-19 pandemic. It is important to examine this interaction effect for two reasons.

First, as stated above, people hold multiple identities at different levels (Brewer, 2001; Crenshaw, 1991). When asked who they are, individuals often list various identities (Brewer, 2001). Identities can be a group to which one belongs or roles that are important to people (Brewer, 2001). Different identities may be associated with competing interests and conflicting values and influence the way people think about issues (Klar, 2013). Nevertheless, most of the scholarly work on identity effects overlooks the competition between identities at different levels. One notable exception can be seen in Klar’s (2013) work. In her study, Klar (2013) examined how individuals reconcile conflicting identity interests when facing multiple identity primes and form attitudes. She found that the effects of a partisan identity prime and a parental prime canceled each other out and did not impact beliefs and attitudes.

Second, because group-based identities have such powerful impacts on attitudes toward controversial scientific issues, it is important to identify how messages might attenuate political polarization on these issues. Based on the discussions in previous sections, this study hypothesizes that linking the COVID-19 pandemic to parental identity would influence parents’ interpretations of factual information related to COVID-19, and further mitigate polarized responses that result from political partisanship. Hypothesis 4 is stated formally as follows:

*Hypothesis 4 (H4):* The parenthood prime is a contributory moderator of the relationship between partisan identity and risk perceptions (H4a), policy support (H4b), social distancing (H4c), and mask-wearing (H4d), such that the effects of partisan identity on these attitudinal outcomes are weaker for parents in the parenthood prime condition.

## Method

### Participants

The data for this study were gathered from 237 participants from a national online Qualtrics panel.^1^ Qualtrics invited panelists who have children under the age of 18 years to participate in this study. Quotas for key demographic variables such as gender, age, and race were targeted to the U.S. Census. Participation in the survey experiment was limited to parents who are U.S. citizens over 18 years old. Participant demographic information is presented in Table 1. Randomization across conditions was successful with no significant differences in key sociopolitical demographic variables.^2^

**Table 1.** Sociopolitical Demographic Makeup of Sample.

Characteristics	*N* (%) or *M* (*SD*)
Gender	
Male	115 (48.5%)
Female	122 (51.5%)
Age (years)	
18–24	28 (11.8%)
25–34	34 (14.4%)
35–44	50 (21.1%)
45–54	39 (16.5%)
55–64	45 (18.9%)
65+	41 (17.3%)
Education	
Some college education or more	180 (75.9%)
Income	
Less than US$25,000	37 (15.6%)
US$25,001–US$50,000	60 (25.3%)
US$50,001–US$75,000	43 (18.1%)
US$75,001–US$100,000	41 (17.3%)
US$100,001–US$200,000	41 (17.3%)
Over US$200,000	15 (6.3%)
Race	
White	170 (71.7%)
Black	30 (12.7%)
Asian American	19 (8.0%)
Other racial identities	18 (7.6%)
Hispanic	29 (12.2%)
The number of children	
1	76 (32.1%)
2	96 (40.5%)
3 or more	65 (27.4%)
Political ideology (7-point scale, “very conservative” coded high)	4.40 (1.92)
Political interest (7-point scale, “very interested” coded high)	5.05 (1.71)
Political knowledge (7-point scale, “very knowledgeable” code high)	4.71 (1.74)
Media use (the number of hours using some form of media on an average day)	8.5 (6.13)

### Procedure

The study was conducted from August 26 to August 28, 2020. Although reports of new cases had dropped considerably nationwide as of August 28, there was an average of more than 42,000 news cases and more than 1,000 coronavirus deaths per day from August 26 to August 28 (Johns Hopkins Coronavirus Resource Center, 2020a). In fact, 12 states underwent increases in their numbers of newly reported cases in August (Johns Hopkins Coronavirus Resource Center, 2020a).

**Figure 1.**
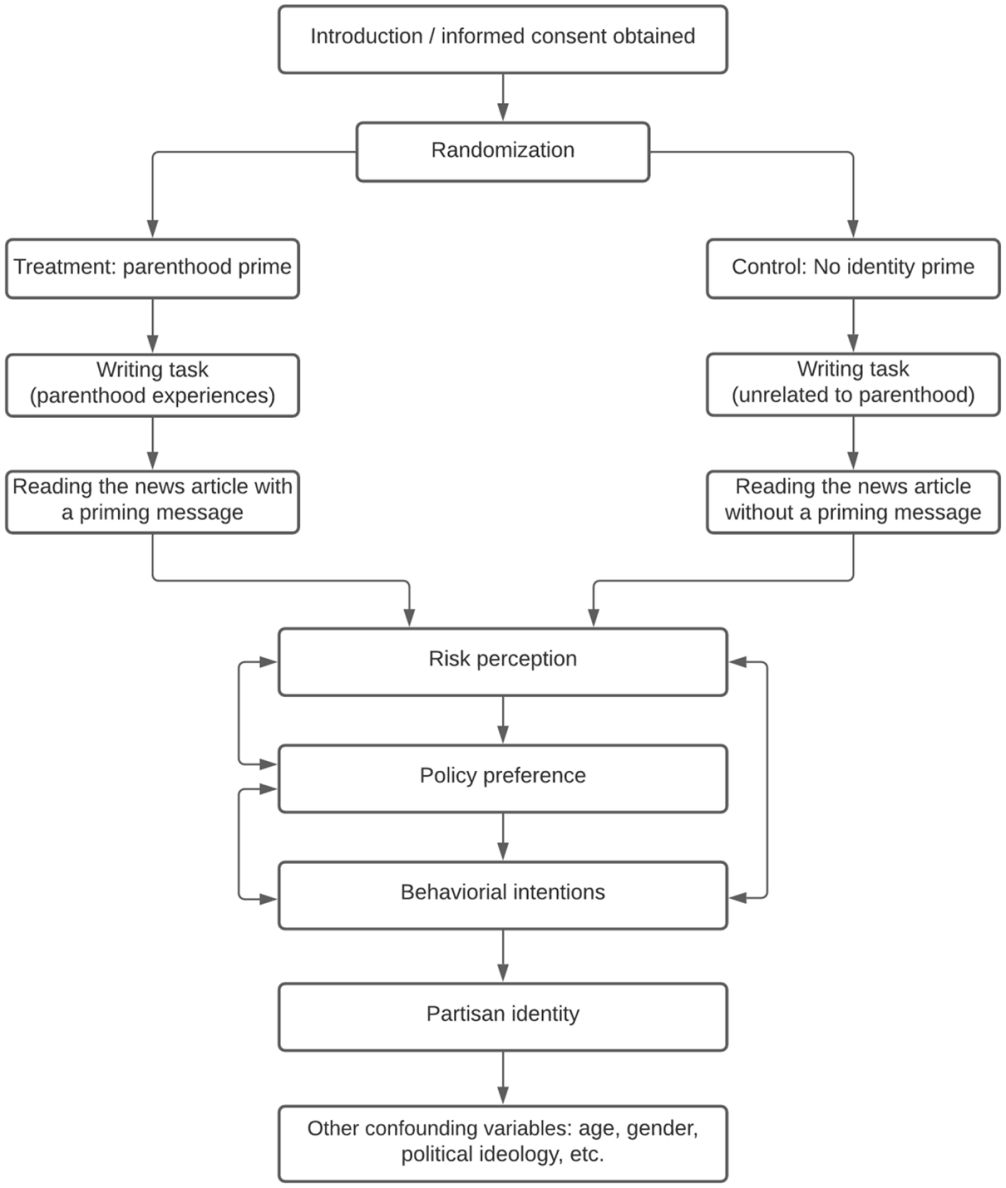
Survey flowchart.

A between-subject design was used in this study. Participants were informed that the study would be about how people make sense of media, information, and society. After consenting to participate in the study, participants were randomly assigned to either a parenthood prime treatment group or a no-prime control group. In the parenthood prime condition, participants were primed with their parental identities by completing a priming task, which included answering two open-ended questions about their experiences of parenthood. Following the priming task, participants read a mock news article containing several basic facts about COVID-19 and precautions to take against the spread of COVID-19. After reading the mock news article, participants in the treatment group were also presented with a short message containing a parental identity cue that was absent from the control condition. Then, they were asked to answer the survey questions. In the control condition, participants were asked to complete a writing task unrelated to parenthood. Then, they were asked to read the same news article and complete the survey questionnaire (see Figure 1 for the survey experiment flow).

### Stimuli

#### Identity Priming

Identity salience was manipulated using two established methods—the basic prime and the efficacy prime (see supplementary material)—from past research. Participants in the parenthood prime group (*n* = 120) were asked to answer two open-ended questions about their experiences of parenthood, for example, “what are three words that characterize your relationship with your child?” After reading the mock news article, participants were presented with the following message: “You have read some basic facts about COVID-19; now we are interested to know what you think about COVID-19 as a *parent*.” Participants in the no-prime control condition (*n* = 117) were asked to list everything that they had eaten and drank in the past 12 hours and then read the same mock news article. Next, participants in the control condition were presented with a version of the short message without the identity cue: “You have read some basic facts about coronavirus (COVID-19); now we are interested to know what you think about coronavirus (COVID-19).”

#### News Article

The simulated news article did not include any identity cues (see Supplementary Material). It contains basic facts about COVID-19 that were published on the CDC (2020b) website.

### Measures

#### Risk Perception

Risk perceptions about COVID-19 were assessed by asking participants about the degrees to which they agreed with five statements, such as “Coronavirus (COVID-19) is a serious public health threat to you” and “Coronavirus (COVID-19) is not as dangerous as people think” (reverse coded). The reliability of the scale was acceptable (Cronbach’s α = .82). The five items were averaged to form a scale, and the mean overall risk perception was 5.31 (*SD* = 1.43).

#### Policy Attitudes

A total of four items were used to measure policy attitudes related to COVID-19. All items were measured on a 7-point Likert-type scale, ranging from 1 (*strongly disagree*) to 7 (*strongly agree*). One item was used to measure participant attitudes toward implementing mass testing: “There should be mass testing for coronavirus (COVID-19).” The remaining items were used to measure participant attitudes regarding COVID-19 restrictions: “The country should be shut down economically because of coronavirus (COVID-19),” “The economic costs of the coronavirus restrictions (e.g., closing schools and businesses) are greater than the benefits to public health (reverse coded),” and “State government should lift all coronavirus-related restrictions on public activities as soon as possible (reverse coded).” The four items were averaged to create a scale (*M* = 4.51, *SD* = 1.24), and the reliability of the scale was acceptable (Cronbach’s α = .62).

#### Intention to Practice Social Distancing

To measure participants’ intentions to comply with social distancing mandates, participants were asked about their likelihoods of practicing the following public health protocols: (a) “Avoid going to events with large crowds, such as concerts, festivals, or sporting events,” (b) “Avoid public places like retail stores and restaurants,” and (c) “Avoid small gatherings of people, such as with friends.” All items were rated on a 7-point Likert-type scale, ranging from 1 (*extremely unlikely*) to 7 (*extremely likely*). The three items formed a scale with a strong level of reliability (*M* = 5.41, *SD* = 1.62, Cronbach’s α = .84).

#### Intention to Wear Face Masks

Participants stated their intentions to wear face masks in a single item (*M* = 6.29, *SD* = 1.34): “How likely are you going to wear a mask or cloth face covering that covers your nose and mouth in public settings in light of the coronavirus (COVID-19) outbreak?” (1 = *extremely unlikely*, 7 = *extremely likely*).

#### Partisan Identity

Partisan identity was measured using a set of standard ANES questions. Based on this set of items, participants in this study were assigned values from 1 to 7. The values were labeled as strong Democrat (*n* = 81), weak Democrat (*n* = 28), Democrat leaner (*n* = 7), pure independent (*n* = 25), Republican leaner (*n* = 11), weak Republican (*n* = 19), and strong Republican (*n* = 66).

#### Sociodemographic and Political Variables

This study included a range of sociodemographic and political variables such as gender, age, education, income, race, residence, and general political knowledge. In addition, confounding variables that might affect the outcome variables being studied were measured, including knowledge about COVID-19, health status, trust in science, and media use.

### Data Management and Analyses

As an attention check, participants were asked to identify the topic of the news article they read. All participants in this study correctly identified the topic of the news article, suggesting high attention to the stimuli. In addition, participants’ responses to the identity priming tasks were checked to determine whether they paid attention to the tasks. No participant gave empty or nonsensical answers. Univariate outliers for all variables were checked. The check indicated that there was one univariate outlier in the number of children variable. This outlier was replaced with a system missing. The response was retained for the analyses because all the other values were acceptable. All the items that were utilized in this study retained less than 1% missing values. In this circumstance, the probability of the missing pattern being missing not at random was extremely low (Howell, 2007). Therefore, listwise deletion was used to deal with the missing data in the analyses.

All stated hypotheses were assessed using the R statistical language. Ordinary least squares (OLS) multiple regression equations were created to understand the effects of partisan identity on attitudes and behavioral intentions related to COVID-19 (H1). The effects of the parenthood prime (H2) were tested through the use of paired-samples *t-*tests. In addition, moderation analyses were conducted using the *Interaction* package in R to explore how gender interacts with the parental identity prime in shaping attitudes (H3), as well as the role of the parental identity prime in mitigating polarized responses to COVID-19 (H4).

## Results

### The Effects of Partisan Identity

H1a through H1d predicted that individuals’ partisan identities shaped their risk perceptions about COVID-19 (H1a), support for restrictive policies to control the outbreak (H1b), and intentions to practice social distancing (H1c) and wear face masks (H1d). OLS multiple regression equations were created to test these predictions. The respective dependent variables were risk perception, policy support, intentions to practice social distancing, and intentions to wear face masks. A total of 12 relevant covariates (e.g., demographics, trust in science, and COVID-19 knowledge) were included in this series of regressions^3^ because each of these variables has been shown to predict attitudes toward COVID-19 or other science-related issues in general (e.g., Ho et al., 2008; Pedersen & Favero, 2020).

As can be seen in Table 2, partisan identity was a significant predictor of risk perceptions about COVID-19 (unstandardized *B* = −.12, *SE* = .04, *p* = .003) and support for restrictive policies (unstandardized *B* = −.09, *SE* = .03, *p* = .006). The results suggested that relative to Democrats, Republicans had lower levels of perceived risk of COVID-19 and were less likely to support restrictive government policies to combat the virus. Concerning the participants’ behavioral intentions to practice social distancing and wear face masks, partisan identity did not have significant impact’s on either behavioral intention. Therefore, only H1a and H1b were supported.

**Table 2.** Multiple Regression Results.

	Risk perception	Policy support	Social distancing	Mask-wearing
Variables	*B* (*SE*)	*B* (*SE*)	*B* (*SE*)	*B* (*SE*)
Partisan identity (Republican)	−.12 (.04)**	−.09 (.03)**	−.02 (.05)	.01 (.04)
Age	.04 (.05)	−.05 (.05)	−.05 (.07)	.08 (.06)
Gender (male)	−.40 (.21)	−.40 (.18)*	−.39 (.28)	−.34 (.22)
Black	.32 (.27)	.51 (.22)*	.58 (.36)	.26 (.28)
Hispanic	−.06 (.26)	−.08 (.22)	−.01 (.35)	.07 (.27)
Education	.10 (.05)	.12 (.05)*	.14 (.07)	.04 (.06)
Income	−.06 (.06)	−.06 (.05)	.03 (.08)	.04 (.07)
Health status	.06 (.06)	−.01 (.06)	.18 (.08)*	.14 (.07)*
Trust in science	.24 (.06)***	.25 (.06)***	.24 (.09)**	.18 (.07)**
Ideology (Conservative)	−.04 (.05)	−.03 (.04)	.00 (.07)	.01 (.05)
Media use	00 (.01)	.02 (.01)	.01 (.02)	−.03 (.01)
Knowledge about COVID-19	.23 (.06)***	.13 (.05)	.10 (.08)	.21 (.08)**
Case level (cases are increasing)	−.05 (.16)	.07 (.14)	−.13 (.21)	−.15 (.17)
*R* ^2^	.37	.37	.13	.21
*F*	10.11***	10.26***	2.52**	4.47***

*Note. B* = unstandardized coefficient; COVID-19 = Coronavirus Disease 2019.

* *p* < .05. **p < .01. ***p < .001.

### The Effects of the Parental Identity Prime

H2a through H2d posited that participants exposed to the parenthood prime had higher levels of risk perception about COVID-19 (H2a), greater support for restrictive policies to control the outbreak (H2b), greater intentions to practice social distancing (H2c), and greater intentions to wear masks in public places (H2d). As shown in Table 3, no significant difference was detected between the control group (*M* = 5.27, *SD* = 1.40) and the treatment group (*M* = 5.34, *SD* = 1.46) concerning the participants’ levels of perceived risk about COVID-19, *t*(235) = −0.34, *p* = .37. Therefore, H2a was not supported. Turning to H2b, a paired-samples *t-*test showed that there was a significant difference in support for restrictive policies to control the pandemic, *t*(235) = −0.80, *p* = .03, supporting H2b. Compared with those in the control condition (*M* = 3.65, *SD* = 1.17), participants in the parenthood prime condition (*M* = 4.09, *SD* = 1.13) showed greater preference for stringent policies. Participants in the treatment condition (*M* = 5.53, *SD* = 1.48) were more likely to practice social distancing than those in the control condition (*M* = 5.29, *SD* = 1.76). However, the difference was not significant, *t*(234) = −1.10, *p* = .27. Similarly, there was no significant difference in the intentions to wear masks between the no-prime condition and the parenthood condition, *t*(235) = −0.87, *p* = .19. Participants in both groups were willing to wear face masks (no-prime condition: *M* = 6.21, *SD* = 1.50; parenthood prime condition: *M* = 6.37, *SD* = 1.16). Therefore, although the general directions of effects might be in line with H2a, H2c, and H2d, there was no statistically significant evidence to support these hypotheses. Only H2b was supported.

**Table 3.** Summary of *t*-Tests for the Two Conditions.

Outcome variables	No prime	Parenthood prime	*t* (*df*)	*p*
	*M* (*SD*)	*M* (*SD*)		
Risk perception	5.27 (1.40)	5.34 (1.46)	−0.34 (235)	.37
Policy attitude	4.05 (1.27)	4.58 (1.19)	−0.80 (235)	.03
Social distancing	5.29 (1.76)	5.53 (1.48)	−1.10 (234)	.14
Mask-wearing	6.21 (1.50)	6.37 (1.16)	−0.87 (235)	.19

### Interaction Between Gender and Parenthood Prime

H3 predicted that in the context of the COVID-19 pandemic, gender was a contributory moderator of the relationship between the parental identity prime and outcome variables including risk perceptions (H3a), policy support (H3b), and intentions to practice social distancing (H3c) and wear face masks (H3d), such that the effects of the parental identity prime were stronger for women.

**Figure 2.**
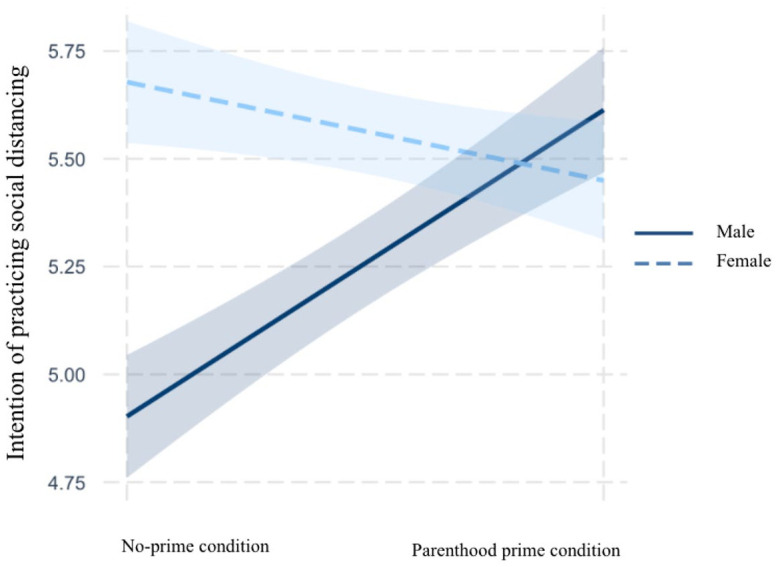
Interaction between parenthood prime and gender on social distancing.

**Figure 3.**
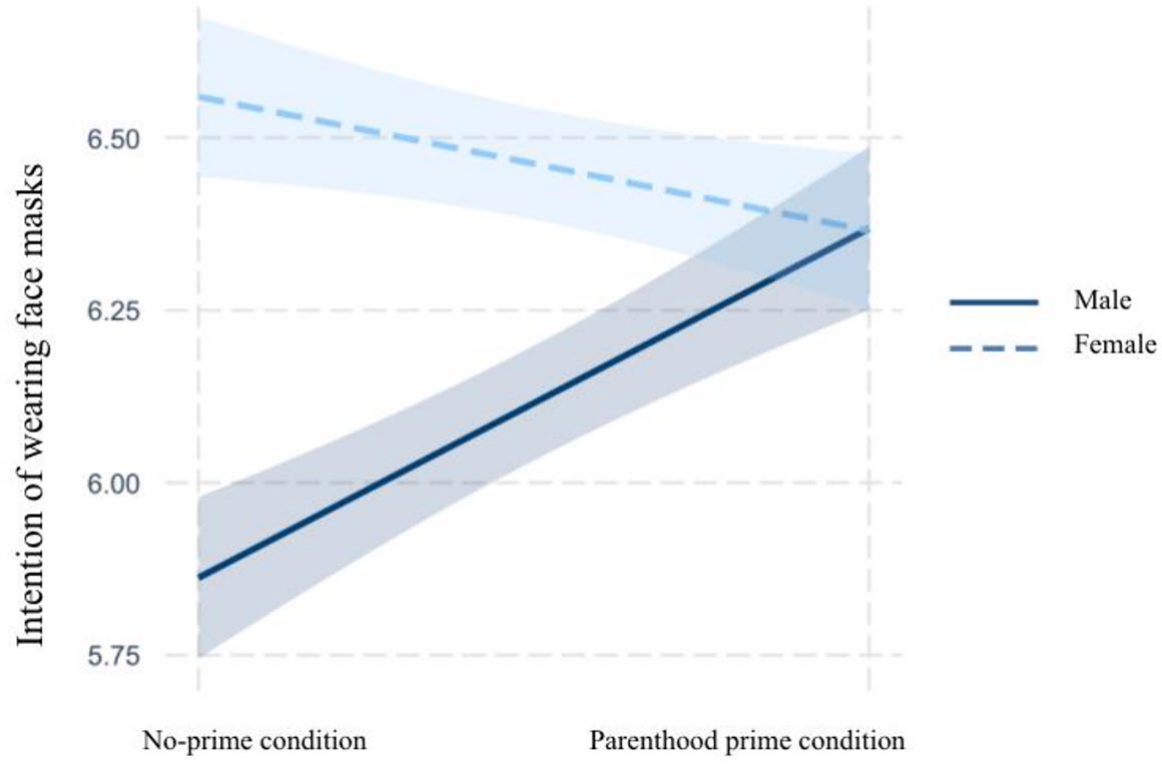
Interaction between parenthood prime and gender on mask-wearing.

As can be seen in Table 4, gender and parenthood did not interact in affecting risk perceptions (unstandardized *B* = .28, *SE* = .36, *p* = .43) and policy attitudes (unstandardized *B* = .22, *SE* = .31, *p* = .47). Therefore, H3a and H3b were not supported. Turning to H3c and H3d, the results suggested that gender significantly moderated the effects of parenthood prime on the participants’ intentions to practice social distancing (unstandardized *B* = .94, *SE* = .42, *p* = .02) and wear masks (unstandardized *B* = .70, *SE* = .34, *p* = .04). However, the directions of the moderation effects were not consistent with the hypotheses. H3c and H3d posited contributory moderations (see Holbert & Park, 2020). However, as can be seen in Figures 2 and 3, gender was a cleaved moderator of the relationship between the parenthood prime and the outcome variables. Specifically, the parenthood prime had positive impacts on intentions to engage in social distancing and wear masks only among fathers. For mothers, the parenthood prime slightly decreased intentions to practice precautious behaviors. Therefore, H3c and H3d were not supported.

**Table 4.** Interaction Between Parental Identity Prime and Gender.

	Risk perception	Policy attitudes	Social distancing	Mask-wearing
Outcome variables	*B* (*SE*)	*B* (*SE*)	*B* (*SE*)	*B* (*SE*)
Parenthood prime	−.09 (.25)	.01 (.21)	−.23 (.30)	−.19 (.24)
Gender (Male)	−.99 (.25)***	−.95 (.22)***	−.78 (.30)*	−.70 (.24)**
Parenthood × Gender	.28 (.36)	.22 (.31)	.94 (.42)*	.70 (.34)*
*R* ^2^	.09***	.12***	.04***	.04***

*Note. B* = unstandardized coefficient.

* *p* < .05. **p < .01. ***p < .001.

### Interaction Between the Parenthood Prime and Partisan Identity

H4a to H4d posited that the parenthood prime interacted with partisan identity in affecting risk perceptions about COVID-19 (H4a), support for restrictive policies to control the outbreak (H4b), and intentions to practice social distancing (H4c) and wear masks in public places (H4d). Moderation analyses were performed to test this set of hypotheses. The results are shown in Table 5. There were significant interactions found between partisan identity and the parenthood prime in predicting perceived risks of COVID-19 (unstandardized *B* = .16, *SE* = .07, *p* = .01), support for restrictive policies (unstandardized *B* = .12, *SE* = .06, *p* = .04), and intentions to practice social distancing (unstandardized *B* = .16, *SE* = .08, *p* = .03). This moderation-based relationship did not exist in predicting intentions to wear masks (unstandardized *B* = .10, *SE* = .07, *p* = .14).

**Table 5.** Interaction Between Partisan Identity and the Parenthood Prime.

	Risk perception	Policy attitude	Social distancing	Mask-wearing
Outcome variables	*B* (*SE*)	*B* (*SE*)	*B* (*SE*)	*B* (*SE*)
Partisan identity	−.47 (.10)***	−.39 (.09)***	−.33 (.13)*	−.22 (.11)*
Parenthood prime	−.59 (.30)	−.35 (.26)	−.40 (.37)	−.24 (.31)
Identity × Parenthood	.16 (.07)*	.12 (.06)*	.16 (.08)*	.10 (.07)
*R* ^2^	.19***	.20***	.04***	.23***

*Note. B* = unstandardized coefficient.

* *p* < .05. ***p < .001.

**Figure 4.**
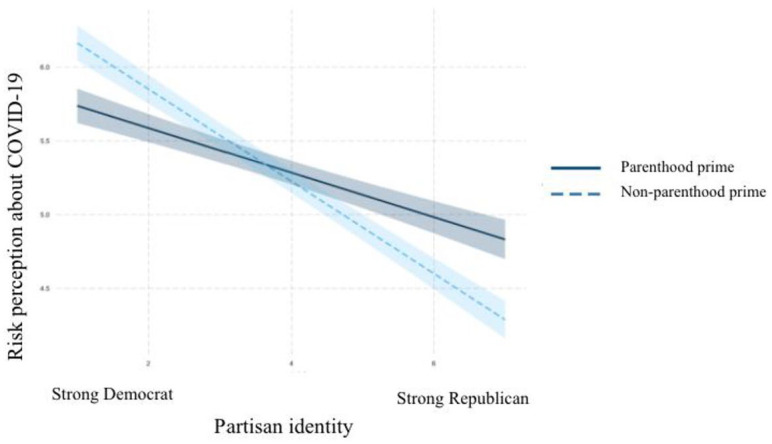
Interaction between partisan identity and parenthood prime on risk perception. *Note.* COVID-19 = Coronavirus Disease 2019.

**Figure 5.**
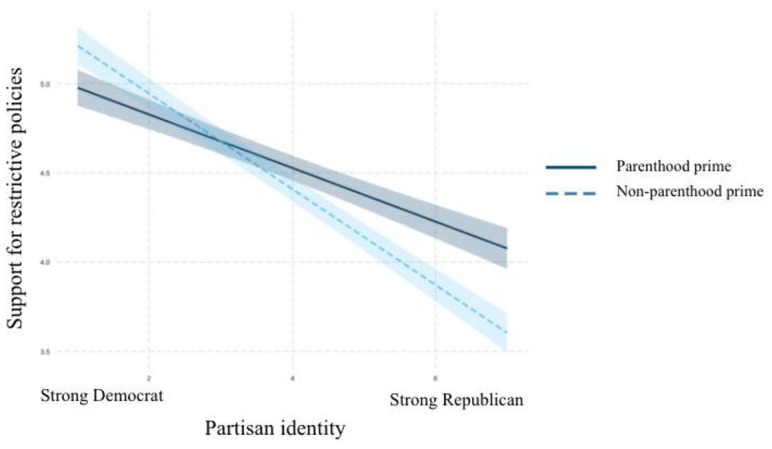
Interaction between partisan identity and parenthood prime on policy support.

**Figure 6.**
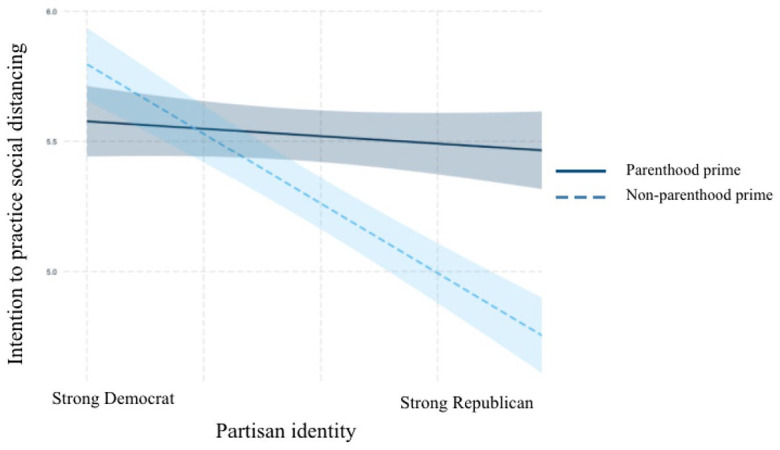
Interaction between partisan identity and parenthood prime on social distancing.

Plotting the interactions in R revealed the existence of the moderation relationships posited in H4a, H4b, and H4c. As can be seen in Figures 4 to 6, among participants primed with their parental identities, partisan identity had less effect on participants’ risk perceptions about COVID-19, support for restrictive policies to control the outbreak, and intentions to practice social distancing than it did among those in the control condition. For the outcome of practicing social distancing, Republicans primed with their parental identity almost held the same levels of intention of engaging in social distancing behaviors as their Democratic counterparts. These results support H4a, H4b, and H4c.

### Additional Analyses

**Figure 7.**
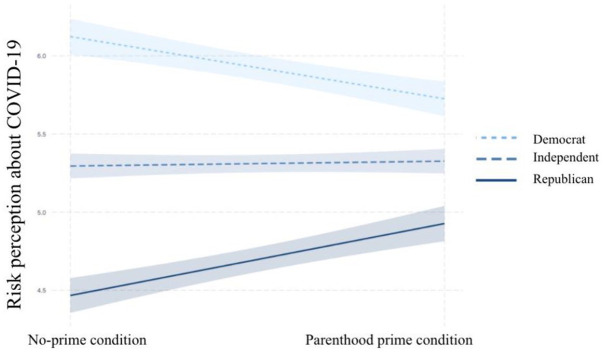
Partisan identity as the moderator in predicting risk perception. *Note.* COVID-19 = Coronavirus Disease 2019.

**Figure 8.**
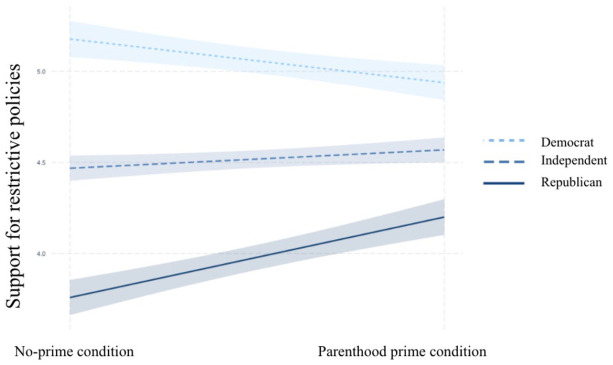
Partisan identity as the moderator in predicting policy support.

**Figure 9.**
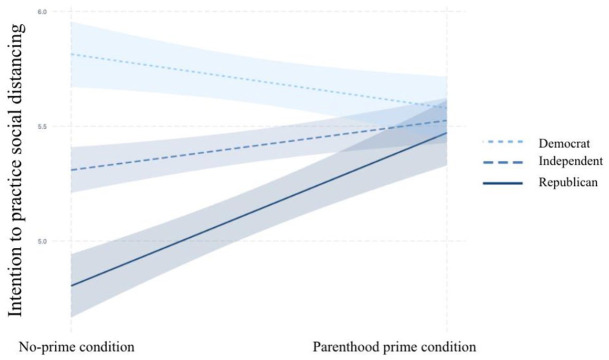
Partisan identity as the moderator in predicting social distancing.

The findings from the moderation analyses discussed above not only suggested that Democrats and Republicans became less polarized when they were primed with their parental identities but also indicated that the parenthood prime had different effects on different people, depending on their partisanships. The interactions were probed to test the conditional effects of the parenthood prime on outcome variables for Democrats, Independents, and Republicans. The pick-a-point technique was used because it can be used to estimate the conditional effect of an independent variable on a dependent variable for any chosen value on the moderator variable scale (Hayes, 2017). The results showed that the parenthood prime only had significant effects on risk perception, policy support, and social distancing for Republican parents (risk perception: *B* = .46, *p* = .04, lower level confidence interval [LLCI] = 0.00, upper level confidence interval [ULCI] = 0.92; policy support: *B* = .44, *p* = .03, LLCI = 0.04, ULCI = 0.84; social distancing: *B* = .67, *p* = .02, LLCI = 0.09, ULCI = 1.24; 95% confidence). The parenthood prime was found to have no significant effects on the outcome variables for Democrats and Independents. As can be seen in Figures 7 to 9, for parents who identified themselves as Republicans, the parenthood prime significantly increased risk perceptions about COVID-19, support for restrictive policies, and intentions to practice social distancing.

Paired sample *t*-tests were performed on subsamples of Republican participants (see Table 6). Results showed that among Republican parents, the parental identity prime significantly increased risk perceptions about COVID-19, *t*(94) = −1.98, *p* = .03, support for restrictive policies to control the pandemic, *t*(94) = −0.80, *p* = .03, and the likelihood of practicing social distancing, *t*(93) = −2.51, *p* = .01, and mask-wearing, *t*(94) = −1.78, *p* = .04.

**Table 6.** Summary of *t*-Tests for the Two Conditions Among Republicans.

Outcome variables	No prime (Republican *n* = 49)	Parenthood prime (Republican *n* = 47)	*t* (*df*)	*p*
	*M* (*SD*)	*M* (*SD*)		
Risk perception	4.31 (1.29)	4.87 (1.47)	−1.98 (94)	.025
Policy attitude	3.65 (1.17)	4.09 (1.13)	−1.87 (94)	.031
Social distancing	4.64 (1.94)	5.53 (1.46)	−2.51 (93)	.006
Mask-wearing	5.80 (1.70)	6.32 (1.11)	−1.78 (94)	.038

## Discussion

Consistent with previous scholarly work on partisan polarization in the context of science communication, this study showed that partisans were motivated to process factual information about COVID-19 through a partisan lens. Republicans across the treatment and control groups had lower levels of risk perception of COVID-19 than Democrats. This finding suggests that risk perceptions about COVID-19 are not affected by the level of underlying risk but, rather, politically relevant interpretations of the risks. Republicans were also less likely to support restrictive measures to help slow the spread of the virus. Partisanship did not predict social distancing and mask-wearing. This might be due to the timing of data collection and the mandates on mask-wearing starting in July 2020. By August 2020, over 5 million cases of coronavirus had been recorded across the United States according to Johns Hopkins Coronavirus Resource Center (2020b). One study found that the increased cases and mask mandates enacted in late July and August 2020 increased mask-wearing compliance to over 90% in all groups (Haischer et al., 2020).

The parenthood prime was found to have a significant impact on policy support. Participants primed with their parental identities had greater preferences for more stringent policies that were imposed to control the spread of the disease compared with those in the control group. Among Republicans, the parenthood prime led to increased levels of risk perception and greater intentions to practice precautious behaviors, such as social distancing and mask-wearing. In addition, the results showed that in the context of the COVID-19 pandemic, the parental identity prime successfully reduced partisan polarization over risk perceptions, policy support, and social distancing behaviors between Republicans and Democrats. Inconsistent with socialization theory, fathers were more likely to be influenced by the parental identity prime.

Although the mechanism by which the parenthood prime reduced the partisan gap in response to COVID-19 was not fully understood, one potential mechanism behind this concerns the role of moral foundations that are tied to partisanship (Graham et al., 2009). Republicans, who value individual liberty, would be less likely to support government-mandated restrictions than others. The parenthood prime, however, is likely to trigger the care/harm moral foundation among Republicans. After all, caring lies at the heart of one’s parental identity (Cowan et al., 1991). This moral intuition further motivates Republicans, especially Republican fathers, to support restrictive policies and comply with public health advisories in an effort to care and prevent harm for their children. This also potentially explains why Democrats and mothers were not influenced by the parenthood prime. Democrats are more focused on caring than Republicans (Graham et al., 2009). It is also conceivable that mothers, regardless of their party affiliations or political ideologies, are more likely to prioritize caring over other moral foundations because they are considered to be primary caregivers. Therefore, they are likely to have strong voluntary prevention efforts and support policy intervention, leaving little room to change their responses to COVID-19.

This study has several theoretical implications for the advancement of scholarly knowledge in science communication broadly. First, this study introduces the concept of relational identity to science communication research that is grounded in social identity theory and motivated reasoning. The findings of this study point to the importance of relation-based identities in shaping issue attitudes. When an issue-relevant relational identity is salient, it can mitigate the influence of partisan identity on attitudes toward politicized science issues. As such, this study underscores the need to incorporate relational identity in identity-based science communication research and examine the complex interconnections among different identities.

Second, this study provides additional evidence of the distinctiveness of identity salience and identity importance, as well as the interplay between the two concepts in affecting interpretations of factual information and issue attitudes. One of the important findings of this study is that the same message can be interpreted differently depending on a person’s currently activated identity. This finding supports the argument that identity importance and identity salience are two distinctive concepts (Morris, 2013). It is true that identities that are considered to be more important to oneself are more likely to be salient than others. However, the identity that comes to mind readily as self-descriptive when reading a message can be different from identities that have high levels of perceived importance to an individual. It is imaginable that a parental identity is acutely important to most parents. However, in this study, parents interpreted facts and evidence related to the virus based on their party affiliations rather than their parental statuses. To summarize, we should not assume that people will utilize their highest order identity at all times and that parents will automatically think about their children first and foremost when forming attitudes on important sociopolitical issues.

This study has practical value as well. The most important finding in this study is that the partisan gap was smaller among participants primed with their relational identity, in this case, their parental identities, than no-prime participants. The parenthood prime evidently moved the attitudes of Republicans in the same ideological direction as those of Democrats. As such, this study provides a strategic communication solution that can influence people’s interpretations of factual information and, ultimately, impact their beliefs and behaviors. The results of this study can be used to help science communicators, policymakers, and journalists develop effective messages about scientific or public health information, especially politicized ones. In the case of COVID-19 specifically, making one’s parental identity salient or highlighting the connections between the pandemic and an individual’s role as a parent in messages has the potential to encourage parents to support restrictive policies, comply with recommendations, and ultimately, mitigate the impact of COVID-19. This strategy could also be used in various cultural contexts because politically divergent responses to COVID-19 were seen in other countries with polarized opinion ecosystems (e.g., Maher et al., 2020).

The major limitation of this study lies in the evolving nature of the pandemic. The COVID-19 pandemic is a public health issue that is ongoing and rapidly changing. A number of factors might be confounded with the results. First, at the time of data collection, the data and trends about COVID-19 were changing on a daily basis. Accordingly, people’s risk perceptions, policy support, intentions to engage in precautious behaviors can change over time. Yet, this study was conducted at a single point in time. It is possible that stronger results might have been obtained during the early stages of the COVID-19 pandemic. In addition, as an ongoing and highly salient issue, people were likely to closely follow news related to the COVID-19 pandemic. It is highly possible that individuals participated in the study right after reading news articles on this pandemic. Therefore, when interpreting the results of this study, it is important to be conscious of the fact that the participants were likely exposed to other information about the pandemic and that the evolving nature of the pandemic could have complicated the relationships that were examined. Finally, the duration of these priming effects remains a question. The effects of relational identity priming might be short-lived and decay over time, similar to the framing effects (Chong & Druckman, 2007). It is unclear whether the participants held the same attitudes and interpreted factual information in the same ways they indicated they did in the survey days or weeks after the survey experiment. Another limitation is that this study employed a single-item measure for intentions to wear masks, and multiple-item scaling would be more advantageous.

Based on this study’s initial findings, future researchers are encouraged to expand this area of research by testing the effects of relational identity salience on issue attitudes for different relational identities (e.g., being a coworker or being a husband) and various scientific issues. In addition, there is a need to employ an intersectionality perspective to examine the effects of individuals’ identities on information processing and sociopolitical attitudes. Future research could address the following questions: How do individuals use their multiple identities to interpret factual information? If primed with both parental and partisan identities, how would individuals engage with both identities simultaneously? What determines which identity is utilized in a particular moment? Scholars can draw on the intersectionality framework to answer these research questions (Crenshaw, 1991).

This study focused on the role of parental identity in shaping individuals’ attitudes and mitigating partisan polarization in the context of COVID-19 in general. However, parents might respond differently to parental identity cues. For example, the effects of a parenthood prime may vary for parents with different marital or legal custody statuses. Past research has found that changes in marital status impact one’s commitment to their parental role, which is an important aspect of parental identity (see Madden-Derdich & Leonard, 2000). This might further affect how a parent responds to a parenthood prime. Future researchers may also look at how interactions and communicative elements within the parent-child relationship moderate the effects of parental identity on message interpretation and attitude change. For example, parenting styles might play a role in this relationship. Future researchers are encouraged to investigate the role that differences in parenting practices (e.g., authoritarian parenting vs. nurturing parents) may play in the way parents process scientific information (Baumrind, 1991; Lakoff, 2002). Another concept to think about is parental involvement. Socialization theory suggests that it is not parental status but parents’ involvement in raising their children that matters in generating attitude and behavioral changes in family settings (Jennings & Niemi, 1974). Future studies could examine whether the effects of parental identity salience on attitudinal outcomes are stronger for parents who have higher levels of parental involvement. Another potential moderator to consider is the age of children. Parenting practices and concerns associated with young children can be different from those associated with adult children. As such, the age of one’s children may interact with parental identity salience in affecting identity-relevant sociopolitical attitudes.

It is also important to consider the potential boomerang effect of relations identity salience on message processing and issue attitudes. For example, for parents with low levels of trust in science, priming parental identity might lead to decreased risk perception about the virus. Therefore, future research will need to examine the conditions under which relational identity-based persuasive attempts are likely to be effective, and when such strategies may actually boomerang.

In sum, this study presents different effects of partisan identity and parental identity primes on information processing and issue attitudes. It also offers a message strategy in science communication—priming people’s parental identities—that could help debias information processing and decrease attitude polarization. As such, the findings of this study, despite the limitations, contribute to the extant literature on identity-based science communication.

## Supplemental Material

sj-docx-1-scx-10.1177_10755470211036676 – Supplemental material for A Relational Identity-Based Solution to Group Polarization: Can Priming Parental Identity Reduce the Partisan Gap in Attitudes Toward the COVID-19 Pandemic Click here for additional data file. Supplemental material, sj-docx-1-scx-10.1177_10755470211036676 for A Relational Identity-Based Solution to Group Polarization: Can Priming Parental Identity Reduce the Partisan Gap in Attitudes Toward the COVID-19 Pandemic by Chen Zeng in Science Communication

## Notes

1. A post hoc statistical power analysis was performed using GPower. The alpha level for this study was preestablished to be 0.05. Power analysis for the *t*-test revealed strong power for detecting large (0.99) and medium effects (0.97) but low power for detecting small effects (0.33). Power analysis for the regression analysis revealed strong power for detecting large (0.99) and medium effects (0.99) but low power for detecting small effects (0.58).

2. No significant difference between the parenthood prime group and the control group was found for gender, *t*(235) = 0.31, *p* = .75, age, *t*(235) = 0.32, *p* = .75, family income, *t*(235) = 0.36, *p* = .72, education, *t*(235) = 0.67, *p* = .50, race (χ^2^ = 2.49, *df* = 5, *p* = .78), religious affiliation (χ^2^ = 6.23, *df* = 8, *p* = .62), political ideology, *t*(235) = −0.02, *p* = .98, political interest, *t*(235) = 0.73, *p* = .47, political knowledge, *t*(235) = 0.90, *p* = .37, trust in the science community, *t*(235) = −1.50, *p =* .14, health status, *t*(235) = 0.09, *p* = .92, media use, *t*(235) = 0.66, *p =* .51, knowledge about COVID-19, *t*(235) = −0.16, *p* = .88, and working from home status (χ^2^ = 2.34, *df* = 1, *p* = .17). A dichotomous variable was created to categorize people living in states where new cases were decreasing (0) and those living in states where new cases were increasing or mostly the same (1) within a 14-day period from August 14 to August 28, 2020, based on data from Johns Hopkins Coronavirus Resource Center (2020a). No significant differences were found between the control group and treatment group in terms of whether the states they live in had upward or downward trajectories: χ2 = .74, *df* = 1, *p* = .39.

3. The study takes the predictive value of six demographic variables into account: age, gender, race, ethnicity, income, and education. These demographic variables have been shown to predict attitudes toward COVID-19 and other science-related issues (see Ho et al., 2008; Pedersen & Favero, 2020). The following variables (i.e., health status, trust in science, COVID-19 knowledge, case levels, and political ideology) were included because each of these variables has also proven itself to affect COVID-19 related attitudes (Dryhurst et al., 2020; Wnuk et al., 2020; Zanin et al., 2020). An additional set of two variables (i.e., trust in science and media use) was included because of the significant roles they play in shaping public attitudes toward controversial scientific issues in general (see Ho et al., 2008).

## References

[bibr1-10755470211036676] Andersen S. M. Chen S. (2002). The relational self: An interpersonal social-cognitive theory. Psychological Review, 109(4), 619–645. 12374322 10.1037/0033-295x.109.4.619

[bibr2-10755470211036676] Arendell T. (2000). Conceiving and investigating motherhood: The decade’s scholarship. Journal of Marriage and Family, 62(4), 1192–1207.

[bibr3-10755470211036676] Batson C. D. (1994). Why act for the public good? Four answers. Personality and Social Psychology Bulletin, 20(5), 603–610.

[bibr4-10755470211036676] Baumeister R. F. Leary M. R. (1995). The need to belong: Desire for interpersonal attachments as a fundamental human motivation. Psychological Bulletin, 117(3), 497–529. 7777651

[bibr5-10755470211036676] Baumrind D. (1991). The influence of parenting style on adolescent competence and substance use. The Journal of Early Adolescence, 11(1), 56–95.

[bibr6-10755470211036676] Bazzi S. Fiszbein M. Gebresilasse M. (2021). “Rugged individualism” and collective (in) action during the COVID-19 pandemic. Journal of Public Economics, 195, Article 104357.

[bibr7-10755470211036676] Brewer M. B. (2001). The many faces of social identity: Implications for political psychology. Political Psychology, 22(1), 115–125.

[bibr8-10755470211036676] Brewer M. B. Gardner W. (1996). Who is this “we?” Levels of collective identity and self representations. Journal of Personality and Social Psychology, 71(1), 83–93.

[bibr9-10755470211036676] Centers for Disease Control and Prevention. (2020a). CDC COVID data tracker. https://www.cdc.gov/coronavirus/2019-ncov/cases-updates/cases-in-us.html

[bibr10-10755470211036676] Centers for Disease Control and Prevention. (2020b). Coronavirus (COVID-19). https://www.cdc.gov/coronavirus/2019-ncov/index.html 34009769

[bibr11-10755470211036676] Centers for Disease Control and Prevention. (2020c). Information for pediatric healthcare providers. https://www.cdc.gov/coronavirus/2019-ncov/hcp/pediatric-hcp.html

[bibr12-10755470211036676] Chan E. Y. (2021). Moral foundations underlying behavioral compliance during the COVID-19 pandemic. Personality and Individual Differences, 171, Article 110463. 10.1016/j.paid.2020.110463 PMC7577686 33106715

[bibr13-10755470211036676] Chong D. Druckman J. N. (2007). Framing theory. Annual Review of Political Science, 10, 103–126.

[bibr14-10755470211036676] Cowan C. P. Cowan P. A. Heming G. Miller N. B. (1991). Becoming a family: Marriage, parenting, and child development. In Cowan P. Hetherington M. (Eds.), Family transitions (pp. 79–109). Lawrence Erlbaum.

[bibr15-10755470211036676] Crenshaw K. (1991). Mapping the margins: Identity politics, intersectionality, and violence against women. Stanford Law Review, 43(6), 1241–1299.

[bibr16-10755470211036676] Day M. V. Fiske S. T. Downing E. L. Trail T. E. (2014). Shifting liberal and conservative attitudes using moral foundations theory. Personality and Social Psychology Bulletin, 40(12), 1559–1573. 25286912 10.1177/0146167214551152 PMC4858184

[bibr17-10755470211036676] Deaux K. (2001). Social identity. In Worell J. (Ed.), Encyclopedia of women and gender (pp. 1–9). Academic Press.

[bibr18-10755470211036676] Dryhurst S. Schneider C. R. Kerr J. Freeman A. L. Recchia G. van der Bles A. M. Spiegelhalter D. van der Linden S. (2020). Risk perceptions of COVID-19 around the world. Journal of Risk Research, 23(7–8), 994–1006.

[bibr19-10755470211036676] Dunlap R. E. McCright A. M. Yarosh J. H. (2016). The political divide on climate change: Partisan polarization widens in the US. Environment: Science and Policy for Sustainable Development, 58(5), 4–23.

[bibr20-10755470211036676] Elder L. Greene S. (2008). Parenthood and the gender gap. In Whitaker L. D. (Ed.), Voting the gender gap (pp. 119–140). University of Illinois Press.

[bibr21-10755470211036676] Evans J. H. Hargittai E. (2020). Who doesn’t trust Fauci? The public’s belief in the expertise and shared values of scientists in the COVID-19 pandemic. Socius, 6, 1–13.

[bibr22-10755470211036676] Gollwitzer A. Martel C. Brady W. J. Pärnamets P. Freedman I. G. Knowles E. D. Van Bavel J. J. (2020). Partisan differences in physical distancing are linked to health outcomes during the COVID-19 pandemic. Nature Human Behaviour, 4(11), 1186–1197. 10.1038/s41562-020-00977-7 33139897

[bibr23-10755470211036676] Graham J. Haidt J. Nosek B. A. (2009). Liberals and conservatives rely on different sets of moral foundations. Journal of Personality and Social Psychology, 96(5), 1029–1046. 19379034 10.1037/a0015141

[bibr24-10755470211036676] Green J. Edgerton J. Naftel D. Shoub K. Cranmer S. J. (2020). Elusive consensus: Polarization in elite communication on the COVID-19 pandemic. Science Advances, 6(28), Article eabc2717. 10.1126/sciadv.abc2717 PMC7455486 32923600

[bibr25-10755470211036676] Haidt J. (2012). The righteous mind: Why good people are divided by politics and religion. Pantheon Books.

[bibr26-10755470211036676] Haischer M. H. Beilfuss R. Hart M. R. Opielinski L. Wrucke D. Zirgaitis G. Uhrich T. D. Hunter S. K. (2020). Who is wearing a mask? Gender-, age-, and location-related differences during the COVID-19 pandemic. PLOS ONE, 15(10), Article e0240785. 10.1371/journal.pone.0240785 PMC7561164 33057375

[bibr27-10755470211036676] Hart P. S. Chinn S. Soroka S. (2020). Politicization and polarization in COVID-19 news coverage. Science Communication, 42(5), 679–697. 10.1177/1075547020950735 PMC7447862 38602988

[bibr28-10755470211036676] Hart P. S. Feldman L. Leiserowitz A. Maibach E. (2015). Extending the impacts of hostile media perceptions: Influences on discussion and opinion polarization in the context of climate change. Science Communication, 37(4), 506–532.

[bibr29-10755470211036676] Hart P. S. Nisbet E. C. (2012). Boomerang effects in science communication: How motivated reasoning and identity cues amplify opinion polarization about climate mitigation policies. Communication Research, 39(6), 701–723.

[bibr30-10755470211036676] Hayes A. F. (2017). Introduction to mediation, moderation, and conditional process analysis: A regression-based approach. Guilford publications.

[bibr31-10755470211036676] Ho S. S. Brossard D. Scheufele D. A. (2008). Effects of value predispositions, mass media use, and knowledge on public attitudes toward embryonic stem cell research. International Journal of Public Opinion Research, 20(2), 171–192.

[bibr32-10755470211036676] Holbert R. L. Park E. (2020). Conceptualizing, organizing, and positing moderation in communication research. Communication Theory, 30(3), 227–246.

[bibr33-10755470211036676] Howell D. C. (2007). The treatment of missing data. In Outhwaite W. Turner S. P. (Eds.), The SAGE handbook of social science methodology (pp. 208–224). SAGE.

[bibr34-10755470211036676] Jenkins R. (2014). Social identity. Routledge.

[bibr35-10755470211036676] Jennings M. K. Niemi R. G. (1974). Political character of adolescence: The influence of families and schools. Princeton University Press.

[bibr36-10755470211036676] Johns Hopkins Coronavirus Resource Center. (2020a). America is reopening. But have we flattened the curve? https://coronavirus.jhu.edu/data/new-cases-50-states

[bibr37-10755470211036676] Johns Hopkins Coronavirus Resource Center. (2020b). COVID-19 in the USA. https://coronavirus.jhu.edu

[bibr38-10755470211036676] Kahan D. M. Braman D. Gastil J. Slovic P. Mertz C. K. (2007). Culture and identity protective cognition: Explaining the white-male effect in risk perception. Journal of Empirical Legal Studies, 4(3), 465–505.

[bibr39-10755470211036676] Kahan D. M. Braman D. Slovic P. Gastil J. Cohen G. L. (2009). Cultural cognition of the risks and benefits of nanotechnology. Nature Nanotechnology, 4(2), 87–91. 10.1038/nnano.2008.341 19197308

[bibr40-10755470211036676] Klar S. (2013). The influence of competing identity primes on political preferences. Journal of Politics, 75(4), 1108–1124.

[bibr41-10755470211036676] Kunda Z. (1990). The case for motivated reasoning. Psychological Bulletin, 108(3), 480–498. 2270237 10.1037/0033-2909.108.3.480

[bibr42-10755470211036676] Lakoff G. (2002). Moral politics. University of Chicago Press.

[bibr43-10755470211036676] Madden-Derdich D. A. Leonard S. A. (2000). Parental role identity and fathers’ involvement in coparental interaction after divorce: Fathers’ perspectives. Family Relations, 49(3), 311–318.

[bibr44-10755470211036676] Maher P. MacCarron P. Quayle M. (2020). Mapping public health responses with attitude networks: The emergence of opinion-based groups in the UK’s early COVID-19 response phase. British Journal of Social Psychology, 59, 641–652. 10.1111/bjso.12396 PMC7361608 32621294

[bibr45-10755470211036676] Maxouris C. (2020, August 26). Covid-19 child cases in the US have increased by 21% since early August, new data shows. CNN. https://www.cnn.com/2020/08/26/health/us-coronavirus-wednesday/index.html

[bibr46-10755470211036676] Morris R. C. (2013). Identity salience and identity importance in identity theory. Current Research in Social Psychology, 21(8), 23–36.

[bibr47-10755470211036676] Nisbet M. Markowitz E. M. (2014). Understanding public opinion in debates over biomedical research: Looking beyond political partisanship to focus on beliefs about science and society. PLOS ONE, 9(2), Article e88473. 10.1371/journal.pone.0088473 PMC3928253 24558393

[bibr48-10755470211036676] Oakes P. J. Turner J. C. Haslam S. A. (1991). Perceiving people as group members: The role of fit in the salience of social categorizations. British Journal of Social Psychology, 30(2), 125–144.

[bibr49-10755470211036676] Painter M. Qiu T. (2020). Political beliefs affect compliance with COVID-19 social distancing orders. SSRN. https://papers.ssrn.com/sol3/papers.cfm?abstract_id=3569098 10.1016/j.jebo.2021.03.019 PMC9754698 36540423

[bibr50-10755470211036676] Pearson A. R. Schuldt J. P. (2015). Bridging climate communication divides: Beyond the partisan gap. Science Communication, 37(6), 805–812.

[bibr51-10755470211036676] Pedersen M. J. Favero N. (2020). Social distancing during the COVID-19 pandemic: Who are the present and future noncompliers? Public Administration Review, 80(5), 805–814. 10.1111/puar.13240 PMC7280647 32836442

[bibr52-10755470211036676] Reid A. Deaux K. (1996). Relationship between social and personal identities: Segregation or integration. Journal of Personality and Social Psychology, 71(6), 1084–1091.

[bibr53-10755470211036676] Sapiro V. (1983). The political integration of women: Roles, socialization, and politics. University of Illinois Press.

[bibr54-10755470211036676] Scheufele D. A. Corley E. A. Shih T. J. Dalrymple K. E. Ho S. S. (2009). Religious beliefs and public attitudes toward nanotechnology in Europe and the United States. Nature Nanotechnology, 4(2), 91–94. 10.1038/nnano.2008.361 19197309

[bibr55-10755470211036676] Sluss D. M. Ashforth B. E. (2007). Relational identity and identification: Defining ourselves through work relationships. Academy of Management Review, 32(1), 9–32.

[bibr56-10755470211036676] Tajfel H. (1981). Human groups and social categories: Studies in social psychology. Cambridge University Press.

[bibr57-10755470211036676] Turner J. C. (1985). Social categorization and the self-concept: A social cognitive theory of group behavior. In Lawler E. J. (Ed.), Advances in group processes: Theory and research (Vol. 2, pp. 77–121). JAI Press.

[bibr58-10755470211036676] Veenstra A. S. Lyons B. A. Fowler-Dawson A. (2016). Conservatism vs. conservationism: Differential influences of social identities on beliefs about fracking. Environmental Communication, 10(3), 322–336.

[bibr59-10755470211036676] Willis H. Janes C. Cha A. (2020, August 10). Children and the virus: As schools reopen, much remains unknown about the risk to kids and the peril they pose to others. The Washington Post. https://www.washingtonpost.com/health/children-and-the-virus-as-schools-reopen-much-remains-unknown-about-the-risk-to-kids-and-the-peril-they-pose-to-others/2020/08/09/e40f0862-d81e-11ea-930e-d88518c57dcc_story.html

[bibr60-10755470211036676] Wnuk A. Oleksy T. Maison D. (2020). The acceptance of COVID-19 tracking technologies: The role of perceived threat, lack of control, and ideological beliefs. PLOS ONE, 15(9), Article e0238973. 10.1371/journal.pone.0238973 PMC7485859 32915908

[bibr61-10755470211036676] World Health Organization. (2020). WHO Director-General’s opening remarks at the media briefing on COVID-19. https://www.who.int/director-general/speeches/detail/who-director-general-s-opening-remarks-at-the-media-briefing-on-covid-19—11-march-2020

[bibr62-10755470211036676] Zanin G. Gentile E. Parisi A. Spasiano D. (2020). A preliminary evaluation of the public risk perception related to the COVID-19 health emergency in Italy. International Journal of Environmental Research and Public Health, 17(9), Article 3024. 10.3390/ijerph17093024 PMC7246845 32349253

