# Supplementary material for: A Relational Identity-Based Solution to Group Polarization: Can Priming Parental Identity Reduce the Partisan Gap in Attitudes Toward the COVID-19 Pandemic
Source: Sci Commun. 2021 Dec;43(6):687–718. doi: 10.1177/10755470211036676 (PMC8371399; doi:10.1177/10755470211036676)

**Supplementary Material**

**Identity Priming Techniques**

Treatment Group: Parental Identity Prime

Before we continue, we are interested to know what traits or characteristics a good parent should have and how you describe your relationship with your child. What are three traits that a good parent should have?

What are three words that characterize your relationship with your child?

[news article]

You have read some basic facts about coronavirus (COVID-19); now we are interested to know what you think about coronavirus (COVID-19) as a parent.

Control Group

Before we continue, we want to make sure that you are familiarized with this survey tool. Please list everything that you have eaten and drank in the past 12 hours.

[news article]

You have read some basic facts about coronavirus (COVID-19); now we are interested to know what you think about coronavirus (COVID-19).

**Mock News Article**


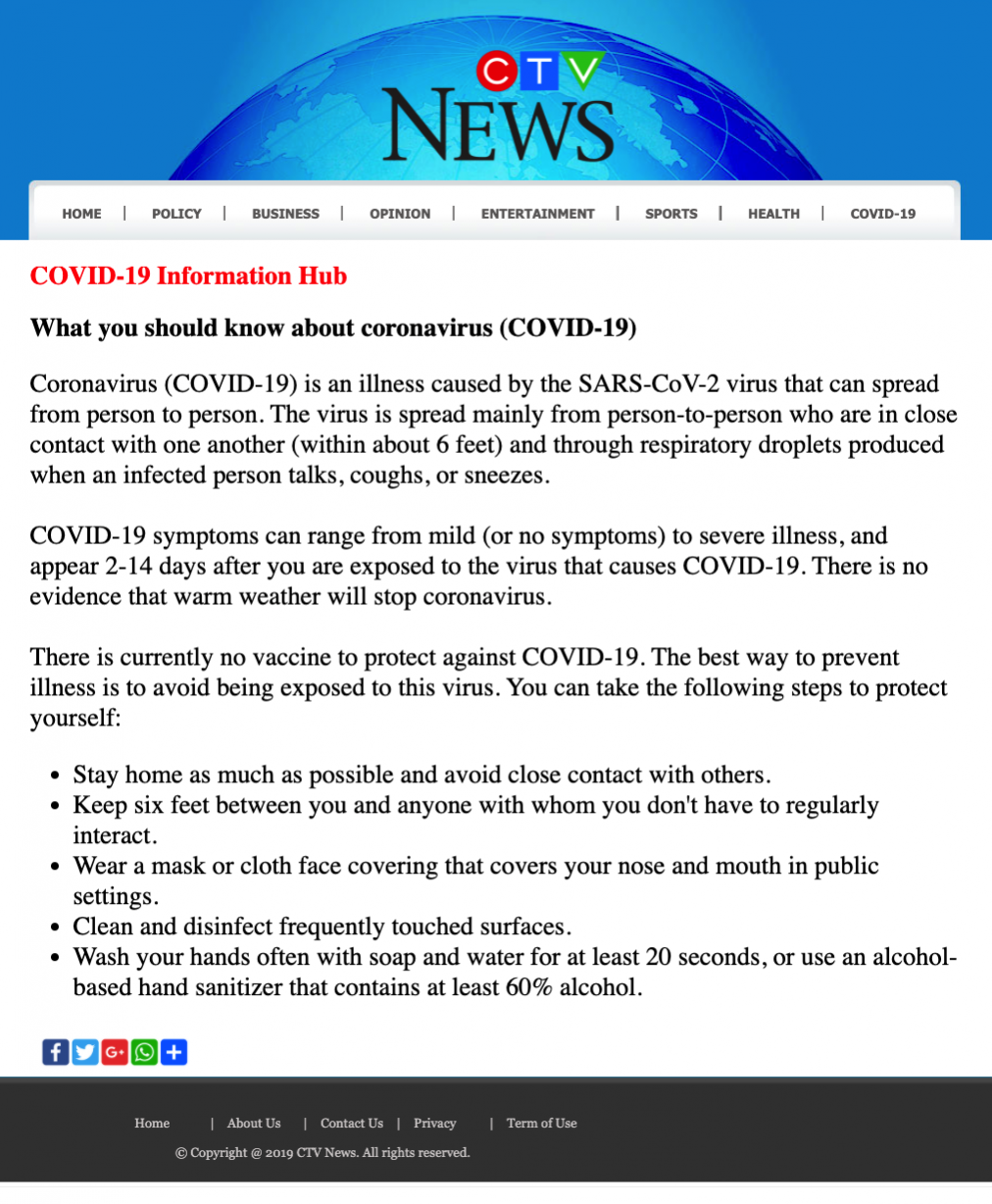

Supplement: sj-docx-1-scx-10.1177_10755470211036676 – Supplemental material for A Relational Identity-Based Solution to Group Polarization: Can Priming Parental Identity Reduce the Partisan Gap in Attitudes Toward the COVID-19 Pandemic [file sj-docx-1-scx-10.1177_10755470211036676.docx]
